# A Case of Actinomyces Prosthetic Hip Infection

**DOI:** 10.7759/cureus.9148

**Published:** 2020-07-12

**Authors:** Sarah N Redmond, Richard Helms, Amanda Pensiero

**Affiliations:** 1 Internal Medicine, Louis Stokes Cleveland Veterans Affairs Medical Center, Cleveland, USA; 2 Internal Medicine, Case Western Reserve University School of Medicine, Cleveland, USA

**Keywords:** actinomyces, prosthetic joint infection

## Abstract

*Actinomyces *is a species of gram-positive anaerobic commensal organisms found in the human oropharynx, gastrointestinal, and genitourinary tracts, which most often are implicated in cervicofacial infections. Rarely do these organisms cause joint infections. We present the case of a 68-year-old man with a prosthetic hip infection due to *Actinomyces *species. He underwent surgical incision and drainage with replacement of the prosthetic joint. Cultures grew *Actinomyces *species, and he was treated with a four-week course of ampicillin-sulbactam followed by eight weeks of amoxicillin-clavulanate. This organism is an uncommon pathogen, and few cases of prosthetic joint infection due to *Actinomyces *have previously been reported.

## Introduction

Hip arthroplasty carries an approximately 2% risk of prosthetic joint infection (PJI) [1]. Typical pathogens include *Staphylococcus aureus*, coagulase negative *Staphylococcus* species, *Streptococcus*, *Enterococcus*, and aerobic gram-negative bacilli. Anaerobic organisms account for approximately 4% of PJIs and *Propionibacterium acnes *is most common. *Actinomyces* species are facultative anaerobic gram-positive bacilli that rarely cause PJI. Review of the literature revealed one case series at a single institution and an additional eight reports of PJI due to *Actinomyces *[2-10]. Among the previously reported cases, infection often occurred years after surgery, both polymicrobial and monomicrobial *Actinomyces* infections were reported, and the majority of reported cases required surgical replacement of hardware followed by antimicrobial treatment with beta-lactam antibiotics [2-9]. Here, we report a case of a patient who developed PJI due to *Actinomyces* six weeks after revision surgery of his prosthetic hip.

## Case presentation

A 68-year-old man presented with left hip pain six weeks after a revision of his left total hip arthroplasty (THA). His original hip replacement surgery was performed 21 years prior to presentation and had required numerous revisions for wear of the joint and one surgery related to infection with methicillin-sensitive *Staphylococcus aureus* (MSSA) 13 years prior. Other notable medical history included uncomplicated right THA and left total knee arthroplasty (TKA) 12 and nine years prior to presentation, respectively, remote history of polysubstance use with alcohol, cocaine, and heroin (treated with suboxone), hepatitis C with sustained virologic response after sofosbuvir/simeprevir, cirrhosis, chronic kidney disease (stage III), coronary artery disease, and hypertension.

The patient’s chief complaint was acute left hip pain, which started two days prior to presentation and progressed until he was unable to bear weight. The patient also noted redness, swelling, small amounts of rust colored drainage from the surgical site, and subjective fever. He denied any recent trauma or contact with dirt or animals to the area. He had been using an unspecified topical cream on the area. He noted occasional oral ulcers but no recent jaw pain, jaw masses, dental procedures, or other complaints. He denied any other joint pain. At the time of presentation, he was febrile to 100.6˚F but hemodynamically stable without tachycardia. Physical exam was significant for two areas of surgical site wound dehiscence of 10 mm without visible purulence but with surrounding erythema, warmth, and fluctuance as well as limited passive hip flexion secondary to pain. Notably, there was no pain on passive internal or external rotation of the joint. His admission labs were notable for a white blood cell count of 9.9 x 1,000/mm^3^, erythrocyte sedimentation rate (ESR) of 114 mm/hr, C-reactive protein (CRP) of >190 mg/L, and serum creatinine of 4.6 mg/dL, slightly elevated from his baseline of 3.2 mg/dL.

Preoperative joint aspiration revealed cloudy fluid with 10,553 nucleated cells (100% neutrophils), and no visible crystals. Gram stain of that fluid was negative. He was started empirically on vancomycin pending cultures. He then underwent incision and drainage with exchange of the head and liner of the joint two days after he first presented. Culture of preoperative joint aspiration fluid and culture of intraoperative tissue biopsies grew *Actinomyces*. The subspecies was not determined but was found to be susceptible to ampicillin-sulbactam with a minimum inhibitory concentration (MIC) of <0.25/0.12 µg/mL by broth microdilution. Notably there was also growth of oxacillin-susceptible *Staphylococcus epidermidis* from broth enrichment of synovial fluid from the preoperative joint aspiration but not from any other specimen. A tunneled catheter was placed, and the patient was started on intravenous (IV) ampicillin-sulbactam and discharged to a skilled nursing facility on postoperative day 8 with a plan to continue IV ampicillin-sulbactam for a four-week course followed by eight weeks of oral amoxicillin-clavulanate, and likely lifelong prophylaxis with amoxicillin-clavulanate.

## Discussion

*Actinomyces* species are facultative, anaerobic, gram-positive bacilli that commonly colonize the human oropharynx, gut, and genitourinary tract as well as skin [8]. Orocervicofacial actinomycosis is the most common infection caused by *Actinomyces* species and manifests as a granulomatous jaw mass [11]. *Actinomyces* species are also implicated less often in bisphosphonate related osteonecrosis of the jaw, pulmonary infections associated with aspiration, pelvic infections associated with intrauterine devices (IUDs), polymicrobial bone and joint infections following exposure of bone, and rarely GI tract infections and brain abscesses thought to be due to hematogenous spread from the lungs or contiguous from cervicofacial infection [8]. These infections are associated with poor oral hygiene, smoking, heavy alcohol use, oral trauma, and dental procedures [11].

*Actinomyces* species rarely cause PJI. Review of the literature revealed 19 cases: 11 cases from a case series at a single large academic institution over 47 years and an additional eight case reports [2-10]. Findings from these cases are summarized in Table 1. Like our patient whose symptoms developed six weeks after his latest revision surgery, patients in the literature presented with late onset of symptoms following either revision or primary joint replacement surgery to that joint, the interval ranging between 20 days and 11 years [2-10]. Comorbid conditions and postulated predisposing factors included obesity, IV drug use, diabetes mellitus, dental procedures, and IUD [2-10]. Notably, the source and route of the infection for our patient remains unclear. He did have a remote history of IV drug use; however, he had remained compliant with suboxone treatment with last reported drug use being many years prior to his current presentation. He also denied any antecedent dental procedures, oral trauma, or lesions. Several cases in the literature report prior infection of the same prosthetic joint with other organisms other than *Actinomyces* [2]. Likewise, our patient had previous infections of that joint many years before which may have predisposed him. It has been theorized that, because prosthetic joints are prone to polymicrobial biofilm formation, clearance of infection is more difficult [1]. However, typically surgical management of PJI requires replacement of hardware [12]. Both polymicrobial and monomicrobial infections with *Actinomyces* species have been reported. Cultures from the above case grew *Actinomyces* with possible *Staphylococcus epidermidis*. However, this was more likely due to contamination as it only grew from one specimen and only after broth enrichment.

**Table 1:** Characteristics of previously reported cases of Actinomyces prosthetic joint infection AMA: against medical advice; BMI: body mass index; COPD: chronic obstructive pulmonary disease; DAIR: debridement, antibiotics, and implant retention; DM2: type 2 diabetes mellitus; GERD: gastroesophageal reflux disease; ICD: implantable cardioverter-defibrillator; IUD: intrauterine device; MI: myocardial infarction; MRSA: methicillin-resistant Staphylococcus aureus; MSSA: methicillin-sensitive Staphylococcus aureus; PAD: peripheral artery disease; PJI: prosthetic joint infection; TMX-SMX: trimethoprim sulfamethoxazole.

Risk factors	Coinfecting organisms	Surgical management	Antimicrobial management	Reference
DM2, BMI>30, smoking, colon surgery, 11 months prior Staphylococcus epidermidis infection to the joint with DAIR	None	Two-stage exchange	Unknown antibiotic	[2]
Smoking, GERD	MRSA, Staphylococcus epidermidis	Two-stage exchange	Six weeks doxycycline	[2]
DM2, prior wound dehiscence with incision and drainage of the joint	Peptostreptococcus, Bacteroides, Staphylococcus epidermidis, Clostridium ramosum	Two-stage exchange	Six weeks vancomycin, ciprofloxacin, and metronidazole	[2]
Prostate cancer with cystoprostatectomy and ileal conduit six years prior, dental procedure seven months prior	Actinobaculum schaalii, Cutibacterium acnes	Two-stage exchange	Six weeks penicillin G and metronidazole followed by four weeks of amoxicillin after implantation	[2]
BMI>30, smoking, prior MSSA infection of joint with two stage exchange three years prior	None	Two-stage exchange	Six weeks ceftriaxone and three months penicillin VK with long-term suppression	[2]
BMI>30, periodontal disease	Peptostreptococcus, MRSA, Enterococcus faecalis	Two-stage exchange	Six weeks vancomycin and ertepenem, then six months amoxicillin with indefinite amoxicillin following reimplantation	[2]
GERD, DM2, PAD, prior hysterectomy, prior PJI with two-stage exchange	Prevotella melaninogenica, Enterococcus, Abiotrophia/Granulicatella, Streptococcus viridans, Pandoraea norinbergensis	Resection arthroplasty not able to reimplant	Six weeks penicillin G and metronidazole, four weeks TMP/SMX followed by six months of amoxicillin	[2]
BMI>30, prior PJI with two-stage exchange	Peptoniphilus, Anaerococcus vaginalis, Streptococcus agalactiae, Trueperella bernardiae, Corynebacterium striatum	Two-stage exchange	Six weeks with vancomycin and ertapenem, lifelong suppression with amoxicillin-clavulanate and doxycycline following reimplantation	[2]
PJI with two-stage exchange four months prior	Staphylococcus epidermidis, Finegoldia magna, Enterococcus faecalis	Two-stage exchange	Six weeks of vancomycin and ertapenem with lifelong suppression with amoxicillin-clavulanate and doxycycline following reimplantation	[2]
Unknown	Finegoldia magna, Cutibacterium acnes, Bacillus	DAIR	Six weeks of ertapenem with lifelong penicillin VK	[2]
IV drug use, Guillain-Barre syndrome complicated by avascular necrosis of femoral head	None	Two-stage exchange	Ampicillin for 20 days before patient left AMA	[3]
DM2, COPD	None	Two-stage exchange	Two weeks of clindamycin and penicillin G followed by six weeks of clindamycin and penicillin G, with two weeks of penicillin G and clindamycin following reimplantation	[4]
Prior MI x 3 with stenting and ICD, Guillain-Barre syndrome	Staphylococcus hominis, Staphylococcus aureus	Incision and drainage of periprosthetic hematoma	Three weeks of amoxicillin and TMX/SMX followed by ofloxacin and rifampicin (duration unknown)	[5]
Dental work several months prior	None	Needle aspiration	Six weeks cefuroxime and rifampin	[6]
Dental prosthesis many years prior	None	Two-stage exchange	Two weeks penicillin G then four weeks amoxicillin	[7]
Dental procedure one month prior	None	Two-stage exchange	Eight weeks of penicillin G, one year of penicillin VK following reimplantation	[8]
IUD infected with Actinomyces	None	IUD removed, patient refused surgery	10 days of penicillin G then patient left AMA	[9]
DM2	None	Two-stage exchange	Six weeks vancomycin then ciprofloxacin and linezolid (duration not reported)	[10]

Typically, treatment of PJI with any organism requires either debridement or resection arthroplasty followed by antimicrobial therapy [12]. Certain cases deemed to be lower risk (symptoms lasting fewer than three weeks, infection occurring within three months of implantation, hematogenous infection, with no evidence of sinus tract) can be debrided with the prosthesis retained. However, more often PJI is managed surgically with one or two-stage exchange of the prosthesis followed by systemic antimicrobial therapy with the length of the course dependent on whether the hardware was retained or exchanged. Patients who are poor surgical candidates can be managed with palliative long-term suppressive antimicrobial therapy. Among reported cases of PJI due to *Actinomyces*, most patients underwent two-stage surgical removal and replacement of hardware followed by antibiotic treatment [2-10]. One patient required resection without replacement due to underlying bone disease, and another patient was able to be debrided with hardware retained [2]. Another patient underwent needle aspiration [6]. The patient in this case underwent single-stage exchange of hardware which is unique among reported cases of *Actinomyces* PJI.

Clinical isolates of *Actinomyces* species are sensitive to beta-lactams. Patients are typically treated with penicillin G or amoxicillin, and less often with ceftriaxone [11,13]. Doxycycline has been used in cases of penicillin allergy [13]. If there is concern for coinfection with beta-lactamase producing organisms such as *Staphylococcus* species, beta-lactam antibiotics combined with beta-lactamase inhibitors (such as amoxicillin-clavulanate or ampicillin-sulbactam) may be used [13]. Oxacillin, cloxacillin, and cephalexin however are not active against *Actinomyces* [11,13]. Additionally, metronidazole and aminoglycosides have little activity against *Actinomyces* in vitro.

After surgical management, most patients in the reported cases of *Actinomyces* PJI were treated with oral or parenteral beta-lactams for six to nine weeks followed by four weeks to one year of suppressive treatment with oral beta-lactams [2,4,8]. Some patients were treated with indefinite suppressive antimicrobial therapy [2]. Specifically, oral penicillin G was most frequently reported; however, certain cases reported ceftriaxone, cefuroxime, ampicillin, and amoxicillin [2-10]. Three patients in the literature underwent treatment with doxycycline [2]. Cases with polymicrobial infection reported use of a wide variety of antimicrobials. Consistent with susceptibilities of clinical isolates of actinomycosis from non-PJI sources, isolates from *Actinomyces* PJI with available susceptibility information were highly susceptible to penicillin G, with several isolates resistant to clindamycin and metronidazole. Breaking from the overall trend of antibiotic use, our patient underwent a four-week course of IV ampicillin-sulbactam followed by an eight-week course of oral amoxicillin-clavulanate with likely long-term suppression with amoxicillin-clavulanate.

## Conclusions

There are few cases of PJI due to *Actinomyces* species reported in the literature. Among the reported cases, proposed risk factors include IV drug use, diabetes, and recent dental procedures. On presentation, patients typically reported joint pain with or without draining sinuses. The reported cases were treated typically with two-stage surgical replacement of the joint followed by antimicrobial therapy with beta-lactam antibiotics. This case demonstrates a patient without a clear source of infection whose PJI was successfully treated with single-stage replacement of his hip arthroplasty and subsequent treatment with combined beta-lactam and beta-lactamase inhibitor antimicrobial therapy. This may be a good choice for PJI in order to cover for possible coinfection with beta-lactamase producing organisms given the tendency for polymicrobial biofilm formation on prosthetic joints. Our case provides additional details to the small body of literature regarding treatment of patients with *Actinomyces* PJI.
